# Executive Functioning Profiles in Neurodevelopmental Disorders: Parent–Child Outcomes

**DOI:** 10.3390/children11080909

**Published:** 2024-07-27

**Authors:** Ana Pardo-Salamanca, Daniela Paoletti, Gemma Pastor-Cerezuela, Simona De Stasio, Carmen Berenguer

**Affiliations:** 1 Department of Developmental and Educational Psychology, Universitat de València, Avda Blasco Ibáñez, 21, 46010 Valencia, Spain; aparsa@alumni.uv.es; 2 Department of Human Studies, LUMSA University, 00193 Rome, Italy; d.paoletti.dottorati@lumsa.it (D.P.); s.destasio@lumsa.it (S.D.S.); 3 Department of Basic Psychology, Universitat de València, Avda Blasco Ibáñez, 21, 46010 Valencia, Spain; gemma.pastor@uv.es

**Keywords:** ASD, ADHD, executive function profiles, parental stress, functional impairment

## Abstract

Background/Objectives: Children with autism spectrum disorder (ASD) and/or attention deficit hyperactivity disorder (ADHD) exhibit more executive function (EF) deficits compared to typically developing (TD) peers. EF deficits are linked to various impairments in daily functioning and increased parental stress. The first aim of the present study is to investigate EFs in children with ASD and ADHD compared to their TD peers. The second aim is to explore profiles of executive functions in children with ASD and ADHD and, finally, to determine the differences of EF profiles in relation to parental stress and children’s functional impairments. Methods: The sample comprised 30 TD children, 47 children with ASD, and 34 children with ADHD, aged 8 to 12 years. Parents completed questionnaires of parenting stress, and children’s social and daily-life functioning. Parents and teachers reported information about children’s EF. Results: The results indicated significantly greater impairment of EFs in the clinical groups compared to the TD group. Moreover, three distinct clusters of functioning were identified based on the severity of reported EF difficulties. The significant findings showed that children with more severe EF profiles were associated with greater daily impairment and higher levels of perceived parental stress. Conclusions: Given the impact of EF deficits on the lives of children with ASD and ADHD and their families, it is crucial that studies like this enhance our understanding and inspire future interventions aimed at improving executive functions in children with ASD and ADHD. Such interventions could help reduce parental stress and improve daily functioning.

## 1. Introduction

### 1.1. Executive Functioning in Children with ASD and Children with ADHD

Executive functions (EFs) refer to a complex set of cognitive abilities such as short-term memory, impulse control, mental adaptability, strategizing, problem solving, and logical thinking [1]. These cognitive control processes are mainly facilitated by the prefrontal cortex, which oversees lower-level processes like perception and motor responses, thus supporting self-regulation and goal-directed behavior [2].

Executive functions (EFs) are vital for cognitive, social, and psychological growth. Research shows that EFs play a role in daily activities like decision making, risk evaluation, planning, prioritizing, sequencing actions, and handling new situations [3]. Therefore, deficits in these domains can significantly impact daily life, leading to inappropriate behavior and impaired social functioning [4]. Executive dysfunction may play a critical role in the outcomes of neurodevelopmental disorders such as autism spectrum disorder (ASD) and attention deficit hyperactivity disorder (ADHD) [5,6,7]. 

ASD is classified as a neurodevelopmental disorder characterized by difficulties in social communication abilities and restricted, repetitive, and stereotyped patterns of behavior [8]. Studies on EFs in individuals with ASD suggest widespread impairments marked by considerable heterogeneity, particularly in cognitive flexibility, planning, organizing, and monitoring [9,10,11,12].

ADHD is also a neurodevelopmental condition, characterized by symptoms in two behavioral areas: inattention and/or hyperactivity–impulsivity [8]. Numerous studies have noted EF deficits in ADHD [13]. Kofler and colleagues [14] highlighted that 89% of children with ADHD showed impairments in executive functioning, and Willcutt et al. [15], in their meta-analysis, stated that individuals with ADHD exhibit significantly greater EF deficits than those without ADHD, specifically in strategizing, spatial and working memory, impulse control, and sustained attention. Despite differences in the manifestations of these disorders, recent meta-analyses have found no differences in EF domains between children and adolescents with ADHD and those with ASD [5,16]. Some researchers propose that the presence of common executive function deficits supports the hypothesis that ASD and ADHD might share overlapping mechanisms [16]. 

However, recent meta-analyses conducted on preschool children with ASD and ADHD from the age of four, compared to TD children, indicated both overlap and specificity in executive function impairments. Shifting was found to be more impaired in ASD compared to ADHD, while inhibition, working memory, and planning were more consistently impaired in ADHD [17].

### 1.2. Executive Function Deficits in ASD and ADHD and Parenting Stress

Raising a child with a neurodevelopmental disorder such as ASD or ADHD, and executive function deficits can be challenging and is considered a risk factor for parental stress [18]. The negative consequences and functional impairments linked to autism or ADHD do not only affect the child; parents of these children also experience significantly higher levels of parenting stress compared to parents of children without that neurodevelopmental condition [19]. 

The literature highlights that there is a direct correlation between parental stress and parental reports of EFs in children with ASD or/and ADHD [20,21]. Behavioral problems exhibited by children with ASD are related to parenting stress [22], and parents who report more stress also tend to report that their children have more EF difficulties [20]. Similar results have been found in parents of children with ADHD. For example, the study conducted by McLuckie et al. [21] found that parent-reported executive dysfunctions accounted for 49% of the variance in child-related parenting stress.

In general, parents of children with ASD or ADHD experience higher levels of stress when their children have greater deficits in EFs, characterized by difficulty in behavioral regulation, impulse control, maintaining and changing attention, and problem solving [20,23]. Among the theories proposed to explain the relationship between EF deficits and parental stress, the transactional development theory is the most appropriate. It underscores the two-way relationship between these constructs, showing that parent and child characteristics interact over time, influencing each other reciprocally [22]. According to this approach, a child’s poor self-regulatory abilities can lead to a parent’s adoption of dysfunctional behaviors and increased stress. At the same time, the parent’s maladaptive behaviors can exacerbate problematic behaviors and further dysfunction in the child [23]. Therefore, such bidirectional influences may result in changes in parental behavior, child behavior, and parent–child interactions. 

In light of the above, it is not so much the type of disorder (ADHD or ASD), but, rather, the behavioral problems related to the child’s EF difficulties that constitute a source of stress for the parents. From the perspective of transactional theory, this stress could, in turn, impact EF weaknesses in the child [20].

### 1.3. Executive Dysfunctions in ASD and ADHD and Children’s Functional Impairments

Deficits in EFs presented by children with ADHD or ASD can lead to functional impairments in different domains of daily life [24,25,26]. Such deficits negatively affect social competence, involving pathways related to theory of mind and social cognition [27,28]. Specifically, difficulties with EFs presented by children and adolescents with ASD may impair their ability to interact effectively with others [25]. The EF’s weaknesses may, therefore, impair ASD children and adolescents’ abilities to develop friendships and maintain quality relationships. On the other hand, friendship quality is able to mediate ASD people’s social impairment, and having low-quality friendships leads to greater difficulties in adjustment [24]. Furthermore, several authors state that from childhood to adolescence, the real-word impairments, related to weaknesses in EFs in individuals with ASD, tend to increase [29].

The literature also highlights similar findings for ADHD children, who often show several functional impairments related to EFs deficits [15,26]. Impairments in EFs domains are associated with generally disruptive [30], uncooperative, and more severe ADHD symptomatology [31]. Considering the impact of EF’s weaknesses on behaviors, they may inevitably also lead to an impairment in the social functioning of children and young people with ADHD. Then, having a good working memory and good higher-order EFs, such as planning, organizing, and strategy making, have been associated with better social skills in young people with ADHD [14,32]. Furthermore, some authors suggest that EF deficits may mediate the link between ADHD and social problems [33]. A recent longitudinal study by Skogli et al. [34] showed the presence of elevated levels of EFs-related daily problems in both ADHD and ASD youth compared to typically developing (TD) youth. 

The authors, however, point to significant differences in developmental trajectories, in that, over the two-year period, daily EF improved for ADHD individuals compared to TD, whereas individuals with ASD showed no improvement compared to TD. Skogli and colleagues [34] suggest that the differences in developmental pathways between the ADHD and ASD group compared to the TD group could be attributed to disorder-specific factors (e.g., main symptoms, treatment) playing a crucial role in the development of daily EFs.

Considering the inconsistent findings on executive deficits profiles in neurodevelopmental disorders such as ASD and ADHD, along with the limited research on the connection between EF and parental stress, this study seeks to fill these gaps in the literature. Most previous studies on ADHD or ASD and parental stress have compared children with these disorders to typically developing children separately. This study, however, is part of a smaller subset of studies [20] that have investigated both clinical groups (ASD and ADHD) of children with EF difficulties and different behavioral profiles, comparing them to typically developing children. Furthermore, unlike most prior studies, this research also examines the potential correlations between EF deficits, parental stress, and children’s difficulties in daily life.

### 1.4. Objectives

The main objective of this study is to explore the EF profiles of children with ASD and children with ADHD thorough the following specific aims:

(i) To analyze the daily EF of children with ASD, and children with ADHD compared with typically developing (TD) peers.
(ii) To explore profiles of children with ASD and children with ADHD, according to EF main indexes (behavioral emotional, cognitive regulation, and global executive composite).
(iii) To determine the differences of EF profiles with perceived parenting stress, and children’s functional impairments.

We hypothesize that profiles of EF characterized by high deficits will be related to more parenting stress and more impairments in daily-life functioning in children with neurodevelopmental conditions.

## 2. Materials and Methods

### 2.1. Participants

This cross-sectional design study is part of an extensive project on parental stress in children with ASD and ADHD. One-hundred and twenty dyads consisting of mothers and their children participated in this study. The children sample was composed of 30 with TD, 47 with ASD severity level 1, and 43 with ADHD, all matched by age and IQ. Children’s ages ranged from 8 to 12 years (mean = 8.40; SD = 1.62) (see Table 1).

Children were enrolled from specialized psychoeducational and clinical health centers. Eligibility required formal diagnoses of ASD without intellectual disability or ADHD by qualified professionals. ADHD children met combined presentation criteria per DSM-IV-TR or DSM-5 (APA) [8,35], with parents and teachers documenting severity on a DSM-5 scale from 0 to 3, including at least six symptoms each of inattention and hyperactivity/impulsivity. ASD children met criteria through assessments like the Autistic Diagnostic Interview—Revised (ADI-R) [36] and the Autism Diagnostic Observation Schedule, Generic (ADOS G) [37], with symptom severity confirmed per DSM-5 and additional assessment through the Social Communication Questionnaire (SCQ) [38]. A minimum IQ of 80 on the Kaufman Brief Intelligence Test 2nd Edition (KBIT-2) [39] was required.

Children with TD were chosen from public schools and showed no signs of psychopathologies or meeting ASD or ADHD criteria as per DSM-5. The exclusion criteria for all groups included an IQ under 80, and any motor, sensory, mental, or neurological impairments. The sample is thoroughly described in Pardo-Salamanca et al. [40].

### 2.2. Measures

#### 2.2.1. Daily Executive Functions

The BRIEF-2 Family Report is a questionnaire that assesses daily executive functions [41]. It contains 63 items that are rated on a frequency scale from “never (1)” to “frequently (3).” The report provides four primary indexes and scores on various executive function-related scales: (a) Behavioral Regulation Index (BRI)—includes inhibition and self-monitoring scales; (b) Emotional Regulation Index (ERI)—includes switching and emotional control scales; (c) Cognitive Regulation Index (CRI)—covers initiative, working memory, planning, organization, and task monitoring scales; (d) Global Executive Composite (GEC)—reflects overall executive functioning. In this study, we utilized the main indexes of the BRIEF-2 Family. Raw scores are converted into age-adjusted T-scores, where a score above 70 indicates clinically significant challenges. The BRIEF-2 Family has demonstrated high internal consistency for the index and composite scores, with Cronbach’s alpha ranges from 0.90 to 0.97 in our sample, which is similar to other studies [41]. The BRIEF-2 exhibits good test–retest reliability with correlation coefficients above 0.80 [41]. The BRIEF-2 Family is an effective tool for identifying executive difficulties in special groups such as the ASD or ADHD populations, demonstrating high internal consistency [42].

*The Conners-3 Teacher Short Form (Conners-3)* [43] was utilized to assess executive functioning. This survey, which is filled out by parents or teachers, assesses behavioral and regulatory disorders in children and teenagers aged 6–18 years. It comprises 39 items categorized into six scales: inattention, learning problems, aggression, family relations, hyperactivity/impulsivity, executive functioning, and peer relations. In this study, particular focus was given to the nine-item Conners-3 Executive Functioning Scale, with good internal consistency (α = 0.87) in our sample. This scale is designed to capture teachers’ observations related to a child’s potential challenges with initiating or finishing projects, tendency to complete tasks at the last minute, and demonstration of inadequate planning, prioritizing, or organizational skills. Teachers assess these aspects using a four-point scale, wherein 0 signifies “never” and 3 signifies “very often”. The resulting T-scores fall within the range of 40 to 90, with lower scores indicative of milder impairments. T-scores are categorized as follows: scores below 40 are considered low, scores between 40 and 59 are average, scores from 60 to 69 are elevated, and scores of 70 or above suggest a very high level of impairment. The Conners-3 Teacher Short-form version demonstrates good internal consistency (α = 0.83–0.91) and test–retest reliability (r = 0.72–0.96) in ADHD groups [43].

#### 2.2.2. Parenting Stress

*The Parenting Stress Index—Short Form (PSI-SF)* [44] is a self-report questionnaire that consists of 36 items and is designed to assess overall parenting stress. It was adapted to Spanish by Díaz-Herrero et al. [45]. The questionnaire is divided into three subscales: parental distress (PD), parent–child dysfunctional interaction (PCDI), and difficult child (DC). Each subscale consists of 12 items, which are rated on a scale from 1 (strongly disagree) to 5 (strongly agree). The total score is obtained by summing the scores of the three subscales, with scores ranging from 36 to 180. A score of 90 or higher may indicate a clinical level of stress. In this study, the three subscales of the PSI-SF completed by the mothers were used. The PSI-SF has demonstrated good psychometric properties, and it is an effective measure for use with high-risk families [46]. The internal consistency for the total scale was high in our sample (Cronbach’s *α* = 0.92).

#### 2.2.3. Children Functional Impairments

*The Weiss Functional Impairment Rating Scale-Parent Form (WFIRS-P)* [47] is a 50-item scale where parents assess their child’s daily functioning over the past month across six different domains: family, school and learning, life skills, child’s self-concept, social activities, and risky activities. Each item is rated on a four-point scale ranging from 0 (“never or not at all”) to 3 (“very often or very much”) or marked as “not applicable”. The average of the scored items for each domain was calculated. This study utilized the subscales of life skills, social activities, and risky activities. The life skills subscale comprises 10 items (e.g., “Excessive use of TV, computer, or video games”, “Keeping clean, brushing teeth, brushing hair, bathing, etc.”, or “Problems with eating (picky eater, junk food)”). The social activities subscale includes 7 items (e.g., “Problems participating in after-school activities (sports, music, clubs)”, “Being teased or bullied by other children”, or “Difficulty with parties (not invited, avoids them, misbehaves)”), and the risky activities subscale consists of 10 items (e.g., “Breaking or damaging things”, “Says mean or inappropriate things”). The scale has been psychometrically validated with an internal consistency Cronbach’s *α* = 0.80 especially in the population with ADHD [48]. For the present study, the internal consistency (Cronbach’s *α*) was 0.78.

*The Strengths and Difficulties Questionnaire (SDQ)* [49] is a widely used tool for assessing a broad spectrum of psychopathological symptoms and prosocial behaviors in children and adolescents between the ages of 4 and 16. This instrument was adapted to the Spanish population by Rodríguez-Hernández et al. [50]. Additionally, it can be completed by either parents or teachers and comprises five subscales: emotional symptoms, hyperactivity/inattention, conduct problems, peer problems, and prosocial behavior. The questionnaire generates a total difficulties score (SDQ total) based on the first four subscale scores, serving as an indicator of the overall level of psychopathological issues in a child. Each subscale offers three response options: not true, somewhat true, and very true, with higher scores indicating more significant difficulties. For the purposes of our study, we focused on the total difficulties score. The SDQ has been found to have satisfactory psychometric properties and reliability, with coefficients ranging from 0.73 to 0.76 [50]. The internal consistency (Cronbach’s *α*) of the emotional and behavioral difficulties subscales for our sample was 0.76 and 0.77, respectively, and it is similar to that reported in a population with special needs [49].

### 2.3. Procedure

This study adheres to the guidelines set forth by the Ethics Committee of the Universitat de València (UV-INV_ETICA-1905517). Parents provided both verbal and written informed consent. The evaluation took place in a room at the Faculty of Psychology. Two trained psychologists administered all the measures to the parents and children.

### 2.4. Data Analysis

Statistical analyses were performed using the Statistical Package for the Social Sciences (SPSS) software version 26.00 (SPSS Inc., Chicago, IL, USA). The distribution of the variables and their conformity to the normal distribution curve were assessed using Kolmogorov–Smirnov tests. To examine the differences in executive functions among the TD, ASD, and ADHD groups, a multivariate analysis of variance (MANOVA) was performed. Further analyses of variance (ANOVAs) were conducted to verify differences across the entire test. For these additional ANOVAs, the significance level was set at *p* < 0.003 following the Bonferroni correction, and the partial eta squared effect size (η^2^_p_) was calculated to determine the strength of the association. A model-based cluster analysis was conducted to identify distinct profiles based on children’s executive function deficits (BRI, ERI, CRI, and the executive functioning scale of the CONNERS-3). Hierarchical cluster analysis was applied using squared Euclidean distance measures and Ward’s minimum variance method to create homogeneous groups, as this approach is more suitable for small sample sizes [51]. Ward’s method is a minimum variance approach used in hierarchical cluster analysis that is generally favored over other techniques, like the single-link method. This method is highly sensitive to outliers and tends to identify clusters of similar size [52]. The subsequent step involved employing k-means analysis, which requires specifying the number of clusters beforehand. The variance ratio criterion (VRC) was used to identify the optimal cluster solution for each chosen cluster. The VRC assesses the ratio of “within variance” (variance explained by the typology) to “between variance”, adjusted for the number of clusters and responses. A three-cluster solution was determined to be optimal based on hierarchical cluster analysis using Ward’s method. This conclusion was also supported by the visual inspection of the dendrogram and the agglomeration coefficients. The clusters were interpreted as representing low, moderate, and high deficits in executive functions. Finally, one-way multivariate analyses of variance (MANOVAs) followed by Tukey post hoc tests were carried out to investigate the effects of varying levels of executive functions among the three clusters on the three subscales of parenting stress and on children’s functional impairments. These impairments included life skills, social and risky activities, and the overall level of social difficulties.

The Tukey post hoc test was chosen because it produces smaller standard errors and increases the likelihood of rejecting the null hypothesis.

The proportion of total variance explained by the independent variables was determined using partial eta squared. According to Cohen [53], the interpretation of eta squared is as follows: less than 0.06 indicates a small effect, 0.06 to 0.14 indicates a medium effect, and greater than 0.14 indicates a large effect.

## 3. Results

### 3.1. EF between Children with TD, ASD, and ADHD 

The MANOVA performed to evaluate the main effect of group on the different variables of executive functions was statistically significant (Wilk‘s Lambda (Λ) = 0.18, F(26,214) = 10.84, *p* < 0.001, η^2^_p_ = 0.57). The confirmation ANOVAs yielded the following results (see Table 2); significant differences (after applying Bonferroni correction) were found for inhibition, F(2,119) = 68.66 *p* < 0.001, η^2^_p_ = 0.54; shift, F(2,119) = 49.44 *p* < 0.001, η^2^_p_ = 0.45; emotional control, F(2,119)= 19.61 *p* < 0.001, η^2^_p_ = 0.25; initiative, F(2,119) = 39.41 *p* < 0.001, η^2^_p_ = 0.40; working memory, F(2,119) = 68.13 *p* < 0.001, η^2^_p_ = 0.53; planning, F(2,119) = 78.87 *p* < 0.001, η^2^_p_ = 0.57; organization, F(2,119) = 38.61 *p* < 0.001, η^2^_p_= 0.39; monitoring, F(2,119) = 61.97 *p* < 0.001, η^2^_p_ = 0.51; self-monitoring, F(2,119) = 77.35 *p* < 0.001, η^2^_p_ = 0.57; BRI, F(2,119) = 84.80 *p* < 0.001, η^2^_p_ = 0.59; ERI, F(2,119) = 70.41 *p* < 0.001, η^2^_p_ = 0.54; CRI, F(2,119) = 90.91 *p* < 0.001, η^2^_p_ = 0.60; and GEC, F(2,119) = 110.60 *p* < 0.001, η^2^_p_ = 0.65.

Tukey post hoc analyses revealed significant differences between the TD group and the clinical groups on all the EF variables analyzed in this study. In addition, scores on the working memory domain and monitoring revealed significant differences between the ADHD group and the ASD group, with higher scores in ADHD children, as Table 2 shows.

### 3.2. Profiles of Executive Function in Children with Autism Spectrum Disorder and Children with ADHD

**Figure 1:**
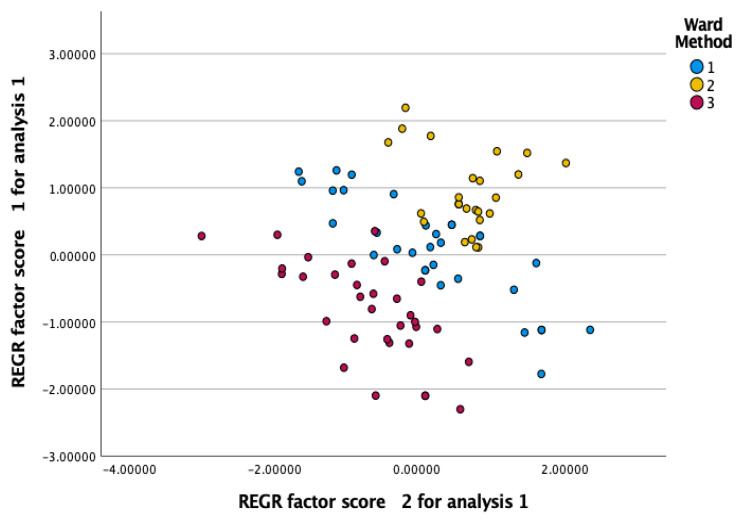
Scatterplot of pairwise comparisons of clusters on executive functioning profiles.

The findings of the hierarchical cluster analysis of the executive functions in children with ASD and children with ADHD are reported in Table 3 and Figure 1. The clusters differentiated executive functioning profiles based on the patterns observed in the scores and were categorized as follows: Profile 1 (n = 32; 35.5%) was categorized as low deficits to reflect low scores on children’s daily executive functioning; Profile 2 (n = 32; 35.5%) was categorized as moderate to reflect average scores on all the EF main indexes and the total scale of executive functioning of the Conners-3; Profile 3 (n = 26; 28.9%) was categorized as high deficits to reflect high scores on the executive functioning in children with autism and children with ADHD.

Analyses of variance (ANOVAs) were conducted to determine the significant differences between the three clusters in the executive function processes.

The results showed statically significant differences between the three profiles. Therefore, the mean global scores for BRI (F = 33.57, *p* < 0.001), ERI (F = 37.74, *p* < 0.001), CRI (F = 39.55, *p* < 0.001), and executive functioning (F = 68.65, *p* < 0.001) were statistically different across the clusters. Furthermore, post hoc comparison showed that Profile 3 (high EF deficits) obtained significantly higher scores than Profile 2 (moderate) and Profile 1 (low EF deficits) among the three main indexes of the BRIEF-2 and the scale of executive functioning of Conners-3.

### 3.3. Differences in Executive Function Profiles with Perceived Parenting Stress, and Children’s Functional Impairments

To address the third study objective, that is, to determine the differences in the executive function profiles and TD children, we carried out analysis of variance. The MANOVA performed to evaluate the main effect of group on the different variables of executive functions was statistically significant (Wilk’s Lambda (Λ) = 0.18, F(21,284) = 10.73 *p* < 0.001, η^2^*p* = 0.43). The confirmation ANOVAs yielded the following results (see Table 4): significant differences (after applying Bonferroni correction) were found for parental distress (PD), F(3,119) = 26.56 *p* < 0.001, η^2^p = 0.43; parent–child dysfunctional interactions (PCDI), F(3,119) = 32.69 *p* < 0.001, η^2^p = 0.48; difficult child (DC), F(3,119) = 80.61 *p* < 0.001, η^2^p = 0.69; life skills (WFIRS-L) F(3,119) = 15.34 *p* < 0.001, η^2^p = 0.30; social activities (WFIRS-S), F(3,119) = 4.69 *p* < 0.001, η^2^p = 0.12; risky activities (WFIRS-R), F(3,119) = 8.44 *p* < 0.001, η^2^p = 0.19; and social difficulties (SDQ-T), F(3,119) = 49.60 *p* < 0.001, η^2^p = 0.58.

Tukey post hoc analyses revealed significant differences between the TD group and the clinical groups on all the variables analyzed in the study; the three scales of parenting stress and children’s functional impairments (life skills, social, risky activities, and social difficulties). In addition, scores on parental distress (PD) did not reveal significant differences between the ADHD group and the ASD group. Scores on parent–child dysfunctional interactions (PCDI), risky activities (WFIRS-R), and social difficulties (SDQ-T) revealed significant differences between Profile 1 (low) and 3 (high). Profiles 1 and 2 showed significantly lower scores than Profile 3 on life skills (WFIRS-L). Profiles 2 and 3 showed significantly higher scores than Profile 1 on social activities (WFIRS-L). Finally, post hoc comparison showed that the three profiles revealed significant differences between them for the scale of difficult child (DC).

## 4. Discussion

The results of this study reveal a significant difference in EF skills between the group of typically developing children and the clinical groups. In fact, the data reveal, for children with ASD and ADHD, deficits in several core domains of EFs, including inhibition, shift, emotional control, planning, organization, monitoring, and working memory skills. In particular, the scores obtained in the working memory and monitoring domains revealed significant differences between the ADHD group and the ASD group, with more severe impairment in ADHD children. These results are in line with previous studies [5,16] that agree that there is greater impairment in EFs in ADHD and ASD children than in TD children.

Moreover, other authors had already highlighted the presence of significant differences between the two clinical groups as well [54,55], but this did not always lead to the same conclusions. For example, in a study by Wang et al. [55], children with ASD reported greater difficulties in visuospatial working memory than ADHD children. On the other hand, however, a study by Happé and colleagues [54] revealed the presence of less severe and persistent EF deficits in ASD than in ADHD; in particular, in planning and monitoring tasks, the ADHD group appeared more impaired than the ASD group. The findings suggest that these differences may be attributable to the different methodologies adopted by the authors to assess these aspects. 

On the other hand, the individual differences observed within the clinical groups allowed the classification of the EFs of ASD and ADHD children into three distinct functioning profiles. The clusters made it possible to distinguish the profiles of EFs on the basis of the scores obtained on the questionnaires administered. Profile 1 is characterized by a lower impairment of the children’s daily executive functioning. Profile 2, on the other hand, reflects a moderate level of impaired executive functioning. Finally, Profile 3 is characterized by more severe deficits in daily executive functioning. Our results show the presence of significant differences between the three clusters in general executive functioning, particularly with regard to the global scores of behavioral, emotional, and cognitive regulation skills, with a greater impairment in Profile 3 than in Profile 2 and Profile 1. Exploring how the different functioning profiles correlated with impairments in other domains of life and parenting stress, this study found significant differences in different domains. Significant differences were recorded between the TD group and the clinical groups for all variables analyzed in the study: the three parental stress scales and the children’s functional impairments (life skills, social, risky activities, and social difficulties). Furthermore, no significant differences in parental distress scores were observed between the ADHD group and the ASD group. Raising a child with a neurodevelopmental disorder requires parents to make an extraordinary commitment and exposes them to higher levels of stress than parents of typically developing children [20]. When in a condition also characterized by the presence of EFs malfunction, the risk of parental stress increases [21]. Indeed, the literature shows that impairments in the EFs of ASD and ADHD children correlate with their parents’ perceived stress [19,20]. It would appear, therefore, that it is not so much the type of disorder (ASD or ADHD) that has an impact on parental stress, but, rather, the behavioral problems related to children’s EFs that foster a condition of parental stress [20].

In particular, the results of this study showed that parents of children with more severe EFs deficits (Profile 3) report higher levels of dysfunctional interactions in the parent–child relationship than those with lesser deficits (Profile 1). Other studies investigating the relationship between EF and parent–child interactions have come to similar conclusions, showing that the quality of perceived interactions between parents and children is significantly associated with performance on executive function tests [56].

Parents of children with low (Profile 1) or moderate (Profile 2) EF impairments attributed better life skills to their children than parents of children with more severe EF impairments (Profile 3). This result is also in line with previous research that recognizes the unique contribution of executive skills in essential aspects of daily life [57]. Impairments in EFs in fact, characterized, for example, by difficulties in inhibition, shift, emotional control, planning, organization, monitoring, and working memory, can impair adaptive behavior and daily living skills [58]. Significant differences were also recorded with regard to social activities, where parents of children with Profile 2 and 3 reported greater impairments than parents with children belonging to cluster 1. Weaknesses in EFs, in fact, can impair the social skills of these children [24], also negatively impacting the quality of relationships [25]. Weaknesses in EFs, in fact, can impair the social skills of these children, also negatively impacting the quality of relationships [25].

Finally, the results of our study also revealed significant differences between the three profiles in relation to parents’ perception of their child as “difficult”. In general, ASD and ADHD children tend to present high levels of negative affect; this, coupled with the presence of deficits in EFs (e.g., control and shift difficulties) [59], could have an impact on parents’ perception of their child’s behavior as “more difficult”.

These findings fit well into the landscape of research investigating executive functions in children with ASD and ADHD, providing insight into how impairments in EFs in this population may be related to parental stress and impairments of daily living.

### Limitations and Further Research

Despite its strengths, this study is not without limitations, such as the moderate sample size, which hinders the ability to draw more generalizable conclusions. Another limitation is the cross-sectional design, which underscores the need for future research involving longitudinal studies. For example, Fossum et al. [60] examined the developmental paths of executive functioning (EF) in individuals with ASD or ADHD, in comparison to typically developing individuals from childhood through young adulthood. The findings revealed that the ASD and ADHD groups generally demonstrated similar levels of maturation on neuropsychological measures and sustained impairments in comparison to the TD group. This suggests the necessity for support of EF in educational, professional, and social contexts during young adulthood for individuals with ASD and ADHD.

Finally, another limitation is the use of self-report questionnaires to assess children’s executive functions. Although these instruments provide valuable information, they may be subject to bias or inaccuracy based on personal perceptions. Future research could include the incorporation of objective measures of EF including, for example, performance-based tasks or neuropsychological assessments to supplement parent/teacher-reported data and provide a more accurate assessment.

Nevertheless, the present study offers interesting insights into the impact that deficits in EFs may have on the daily lives of ASD and ADHD children as well as on parental stress.

Future research could be concerned with exploring potential moderating or mediating variables, such as socioeconomic status, social support, and quality of parenting, which could play a role in the relationship between EFs deficits, daily impairment, and parental stress. This could be useful with a view to structuring interventions aimed at enhancing the EFs of ASD and ADHD children.

## 5. Conclusions

In conclusion, this study reveals significant differences in EF skills between typically developing children and those with ASD or ADHD, highlighting deficits in inhibition, shift, emotional control, planning, organization, monitoring, and working memory among the clinical groups. ADHD children exhibited more severe impairments in working memory and monitoring compared to ASD children. Variations in EF deficits between ASD and ADHD groups were also noted, possibly due to different assessment methodologies. This study classified children into three EF functioning profiles, with Profile 1 showing lower impairment, Profile 2 moderate impairment, and Profile 3 severe deficits. Significant differences in behavioral, emotional, and cognitive regulation skills were observed across these profiles, with higher impairment correlating with greater parental stress and dysfunctional parent–child interactions. Parents of children with severe EF deficits (Profile 3) reported higher levels of stress and poorer life and social skills in their children compared to parents of children with less severe deficits. This suggests that EF-related behavioral problems, rather than the type of disorder, significantly impact parental stress. This study also found that children with better EF skills had better adaptive behavior and daily living skills, and fewer social difficulties. Limitations include the moderate sample size and cross-sectional design, necessitating longitudinal studies to understand EF developmental trajectories in ASD and ADHD. Future research should incorporate objective EF measures and explore moderating factors like socioeconomic status and social support to better understand EF deficits’ impact on daily impairment and parental stress. 

The clinical implications of this study can significantly inform educational strategies and caregiving approaches for children with ASD or ADHD, thereby potentially reducing parental stress. By recognizing and addressing the specific EF deficits highlighted—such as inhibition, emotional control, planning, organization, monitoring, and working memory—educators and caregivers can tailor interventions to better support these children. For instance, targeted training and support in areas like working memory and monitoring, particularly for children with ADHD, can help mitigate some of the challenges they face as inattention or hyperactivity/impulsivity. Additionally, understanding the different EF profiles (lower, moderate, and severe impairments) allows for more individualized and effective intervention plans. Educators can implement classroom strategies that cater to each child’s unique needs, fostering a more supportive and inclusive learning environment. Caregivers can be provided with resources and strategies to enhance their child’s adaptive behavior and daily living skills, thereby reducing the incidence of behavioral problems that contribute to parental stress. Overall, this personalized approach not only supports the child’s development but also alleviates parental stress by improving parent–child interactions and reducing the behavioral and social difficulties associated with severe EF deficits.

## Figures and Tables

**Table 1:** Sociodemographics.

	TD (n = 30)		ASD (n = 47)		ADHD (n = 43)			
	M	SD	M	SD	M	SD	F _(2,119)_	*p*
Age	8.66	1.32	9.37	1.75	9.29	1.38	1.69	0.171
IQ	100.02	8.21	98.37	10.92	98.94	6.91	0.48	0.611
Sex (% male)	65.6%		89.32%		90.72%			
Med (% yes)			40.41%		79.04%			
ASD S.			28.37	5.71				
ADHD S.			24.62	10.73	41.05	9.12		
P. Education level	3.15	0.83	3.05	1.09	1.88	1.40	0.53	0.581

Note. Med (medication), P. Education level (parental education level); parental education level measured as highest level of mother or father (0 = no education, 1 = primary education, 2 = basic secondary education, 3 = advanced secondary education, 4 = university education, ASD S (ASD symptoms (ADI-R)), ADHD S (symptoms; total score DSM-5 parents report); *p* < 0.05.

**Table 2:** Differences between ASD, ADHD, and TD groups in children executive functions.

	TD (n = 30)		ASD (n = 47)		ADHD (n = 43)				
	M	SD	M	SD	M	SD	F _(2,119)_	η^2^_P_	Tukey Post Hoc
Inhibition	50.72	6.33	72.94	9.84	73.61	10.59	68.66 *	0.54	ASD,ADHD > TD
Shift	54.84	8.46	81.36	12.66	76.09	13.38	49.44 *	0.45	ASD,ADHD > TD
Emotional c	53.91	6.96	65.21	11.13	70.84	14.62	19.61 *	0.25	ASD,ADHD > TD
Initiative	52.81	9.27	71.47	10.93	70.88	9.62	39.41 *	0.40	ASD,ADHD > TD
WM	51.31	8.87	70.43	7.88	76.00	11.02	68.13 *	0.53	ADHD > ASD > TD
Planning	49.91	7.44	70.72	9.12	71.70	7.78	78.87 *	0.57	ASD,ADHD > TD
Organization	51.69	6.31	72.38	10.28	70.16	13.89	38.61 *	0.39	ASD,ADHD > TD
Monitoring	50.34	7.63	68.57	9.05	71.86	9.19	61.97 *	0.51	ADHD > ASD > TD
Self-monitor	49.63	5.14	71.68	8.95	70.84	9.79	77.35 *	0.57	ASD,ADHD > TD
BRI	51.21	7.19	74.23	7.54	74.63	11.06	84.80 *	0.59	ASD,ADHD > TD
ERI	54.14	6.54	75.36	9.65	78.23	11.58	70.41 *	0.54	ASD,ADHD > TD
CRI	51.56	6.72	74.63	9.11	76.37	10.05	90.91 *	0.60	ASD,ADHD > TD
EF	53.10	6.12	76.47	11.62	78.09	12.16	110.60 *	0.65	ASD,ADHD > TD

Note. Emotional c (emotional control), WM (working memory), BRI (behavior regulation index), ERI (emotion regulation index), CRI (cognitive regulation index), EF (executive functioning subscale CONNERS-3), * *p* < 0.003 (Bonferroni correction for multiple comparison).

**Table 3:** Executive function profiles in children with ASD and children with ADHD.

	Profile 1 Low (n = 32)		Profile 2 Moderate (n = 32)		Profile 3 High (n = 26)				
	M	SD	M	SD	M	SD	F _(2,87)_	η_p_^2^	Tukey Post Hoc
BRI	66.84	7.40	76.06	6.39	81.85	7.44	33.57 **	0.43	1 < 2 < 3
ERI	67.97	7.71	78.44	9.60	85.77	5.25	37.74 **	0.46	1 < 2 < 3
CRI	68.19	8.27	75.56	7.03	84.58	4.83	39.55 **	0.47	1 < 2 < 3
EF	68.88	5.68	78.78	3.79	84.12	7.26	63.65 **	0.58	1 < 2 < 3

Note. BRI (Behavior regulation index), ERI (Emotion regulation index), CRI (Cognitive regulation index), EF (Executive functioning subscale CONNERS-3), ** *p* < 0.001.

**Table 4:** Analyses of variance (ANOVAs) of parenting stress scales and children’s outcomes among the profiles and TD group.

	TD (n = 30)		Profile 1 Low (n = 32)		Profile 2 Moderate (n = 32)		Profile 3 High (n = 26)				
	M	SD	M	SD	M	SD	M	SD	F _(3,119)_	η_p_^2^	Tukey Post Hoc
PSI-PD	23.67	5.58	37.09	10.38	40.50	7.74	42.12	9.95	26.56 *	0.43	TD < 1,2,3
PSI-PCDI	20.63	4.46	33.59	9.77	39.22	8.00	42.58	21.25	32.69 *	0.48	TD < 1,2,3,1 < 3
PSI-DC	20.97	5.05	38.00	7.88	42.09	6.32	45.73	7.06	80.61 *	0.69	TD < 1,2,3,1 < 2 < 3
WFIRS-L	9.26	4.01	14.00	5.88	14.65	4.50	17.30	4.11	15.31 *	0.30	TD < 1,2,3,1,2 < 3
WFIRS-S	6.93	2.97	7.73	5.26	10.40	4.85	10.86	4.95	4.69 *	0.12	TD < 1,2,3,1 < 2,3
WFIRS-R	2.33	1.32	4.23	3.00	4.90	2.81	6.36	3.79	8.44 *	0.19	TD < 1,2,3, 1 < 3
SDQ-T	8.43	8.15	23.80	6.61	26.88	5.30	32.77	5.12	49.60 *	0.58	TD < 1,2,3,1 < 3

Note. PSI (Parenting Stress Index), PD (parental distress), PCDI (parent–child dysfunctional interaction), DC (difficult child), WFIRS (Weiss Functional Impairment Rating Scale-Parent Form), L (life skills), S (social activities), R (risky activities), SDQ-T (Strengths and Difficulties Questionnaire total score), * *p* < 0.007 (Bonferroni correction for multiple comparison).

## Data Availability

The data presented in this study are available on request from the corresponding author. The data are not publicly available due to privacy.
